# Case Report: A rare successful case of treating cerebral air embolism following the removal of a central venous catheter without intracardiac structural abnormalities

**DOI:** 10.3389/fsurg.2025.1673766

**Published:** 2025-11-21

**Authors:** Mingzhe Song, Wenxiang Wang, Jie Wu

**Affiliations:** 1 The Second Department of Thoracic Surgery, The Affiliated Cancer Hospital of Xiangya School of Medicine/Hunan Cancer Hospital, Central South University, Changsha, China; 2 The Second Department of Thoracic Surgery, Hunan Clinical Medical Research Center of Accurate Diagnosis and Treatment for Esophageal Carcinoma, Changsha, China

**Keywords:** case report, esophageal cancer, cerebral air embolism, central venous catheter, air embolism

## Abstract

**Background:**

Cerebral air embolism following the removal of a central venous catheter is a rare and often fatal complication, resulting in severe neurological deficits and potentially death.

**Case presentation:**

We report a unique case involving a 57-year-old male, where the postoperative removal of a central venous catheter resulted in cerebral air embolism in a patient with esophageal cancer who had no intracardiac structural abnormalities. Following treatment that included low-temperature brain protection and the reduction of cerebral edema, a favorable prognosis was achieved.

**Conclusions:**

This case underscores the efficacy of prompt low-temperature brain protection in managing cerebral air embolism. Additionally, it examines potential mechanisms underlying cerebral air embolism in the absence of intracardiac structural abnormalities.

## Background

The central venous catheter (CVC) is widely utilized for fluid management, drug infusion, and hemodynamic monitoring in critically ill, oncological, and postoperative patients. Despite its clinical value, CVC-related complications, such as infection, thrombosis, catheter malposition, and air embolism, cannot be overlooked. Among these complications, while the incidence of air embolism (AE) is relatively low, ranging from 0.1% to 2%, its occurrence, particularly cerebral air embolism (CAE), can result in severe neurological deficits and potentially death (1–4).

Here, we report a postoperative esophageal cancer patient who self-removed his internal jugular central venous catheter and developed a catheter-related air embolism. He was successfully managed with comprehensive treatment and made a full recovery.

## Case report

**Figure 1:**
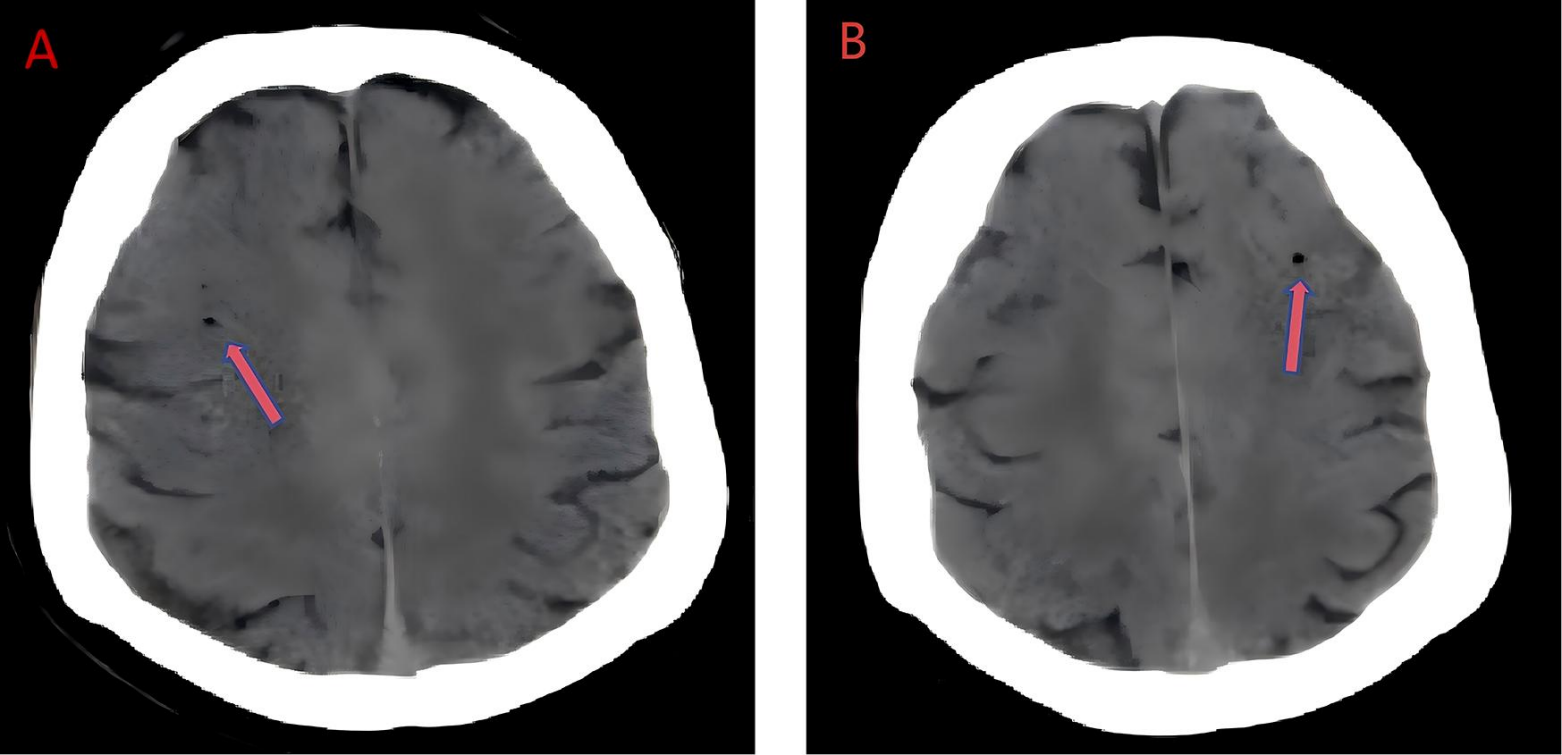
Brain computed tomography **(A, B)** revealed scattered small air shadows in both frontal lobes, suggesting gas embolism (arrows) in both cerebral hemisphere.

**Figure 2:**
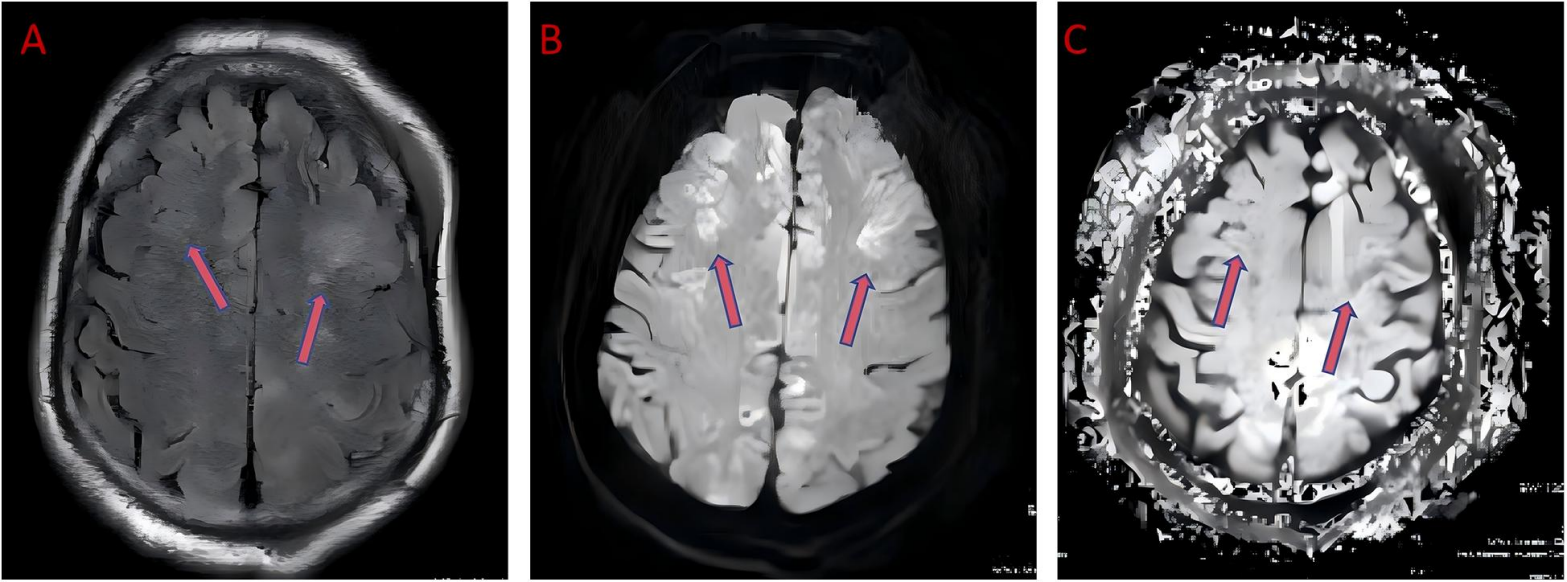
Head MRI shows swelling in the frontal and parietal lobes, indicating ischemic hypoxic encephalopathy. FLAIR imaging shows that brain edema is located in both frontal and parietal lobes, indicating the extent of cerebral ischemia and hypoxia **(A)**,The high signal of DWI sequence indicates ischemic areas in brain tissue **(B)**,The dark signal of the ADC sequence confirms the location of the ischemic and hypoxic regions in the brain **(C)**.

**Figure 3:**
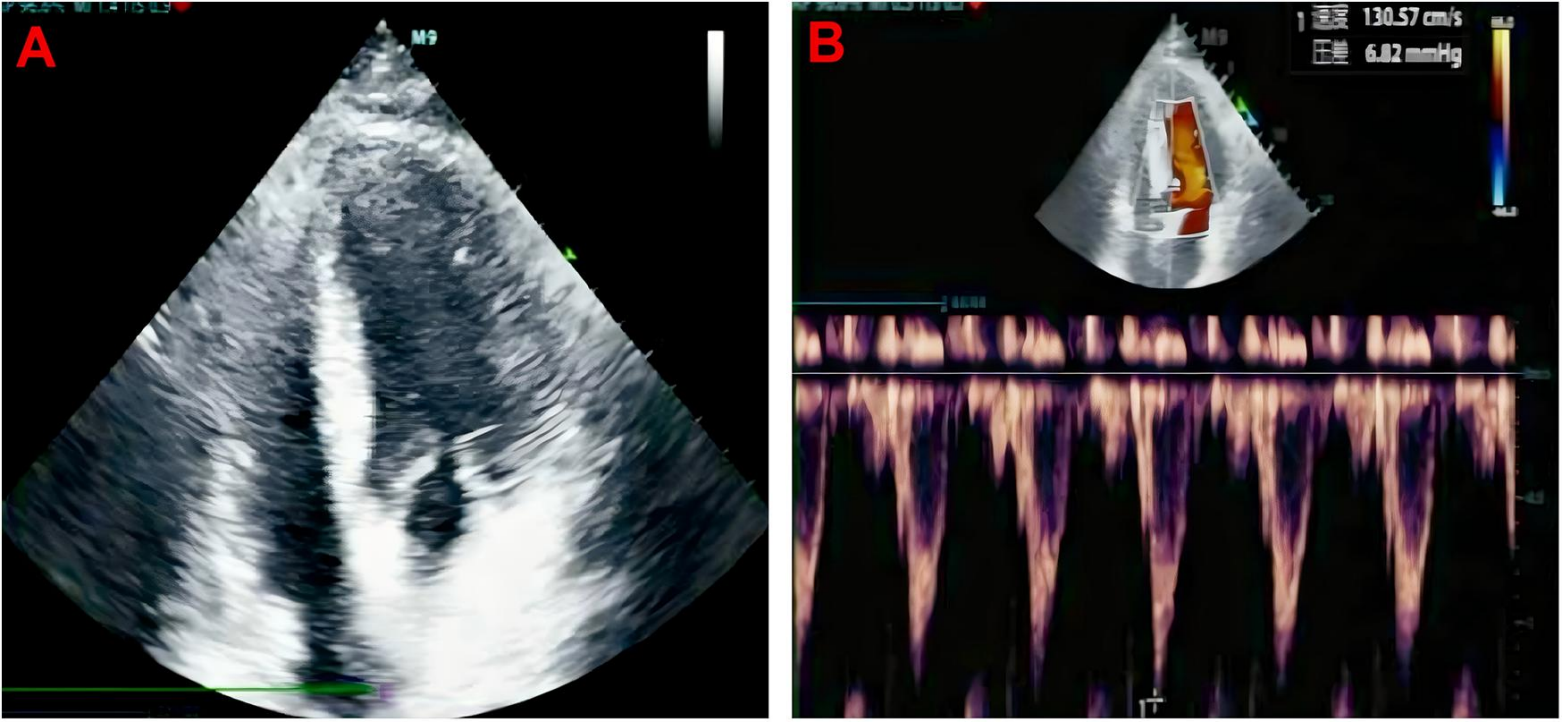
Normal apical four chamber echocardiography **(A)**, normal four chamber aortic valve blood flow spectrum **(B)**. Cardiac ultrasound did not show any structural abnormalities in the heart.

A 57-year-old male patient was diagnosed with lower esophageal squamous cell carcinoma and underwent esophageal cancer radical surgery at our hospital. On the fourth day after the operation, the patient suddenly lost consciousness and had decreased blood oxygen when going to the toilet after removing the Internal jugular vein CVC catheter connector by himself. This was considered CAE according to clinical manifestations and behavior. After performing an emergency tracheal intubation, the anesthesiologist conducted a brain CT scan, revealing scattered small air shadows in both frontal lobes, indicating the presence of gas embolism within a brain blood vessel (Figure 1). CTA of the head and neck showed no vascular abnormalities (Figure 2A). Cardiac ultrasound did not show any heart structural abnormalities (Figure 3). Following the diagnosis of venous air embolism, the patient was immediately placed in a left lateral decubitus and Trendelenburg position. Sedation, hypothermia, and reduced cerebral edema treatment were administered to the patient. The following day, a brain MRI showed swelling in the bilateral frontal and parietal cortex, indicating ischemic and hypoxic brain (Figures 2B,C). Additionally, edema of soft tissue beneath the scalp in the left hemisphere of the brain was noted. At the same time, hyperbaric oxygen therapy was considered. However, it was not administered as the patient remained hemodynamically stable and exhibited rapid neurological improvement with supportive care alone. Thus, we continued sedation with propofol and administered methylprednisolone and mannitol to reduce cerebral edema. After three days, the patient's consciousness recovered; their pupils were reactive to light; upper limb muscle strength returned to normal; and lower limb muscle strength returned to 2–3 levels. Continue treatment for one week and be discharged (Table 1).

**Table 1:** Timeline of clinical events and management.

Date & time (Post-CVC removal)	Clinical event/intervention	Key findings/outcome
Day 1—20:20	Accidental removal of the right internal jugular Central Venous Catheter (CVC).	The patient was sitting upright at approximately 60 degrees.
Day 1—20:32 (Within minutes)	Acute onset of neurological symptoms?	Patient experienced sudden loss of consciousness and desaturation.
Day 1—20:35 (Within 15 min)	Emergency resuscitation initiated.	Transferred to the intensive care unit for tracheal intubation assisted breathing,Fluid resuscitation begun.
Day 1—22:00 (Within 2 h)	Non-contrast Computed Tomography (CT) of the head performed.	CT shows scattered small air shadows in both frontal lobes, suggesting gas embolism.
Day 1—22:30	Transthoracic Echocardiogram (TTE) performed.	TTE showed no evidence of a Patent Foramen Ovale (PFO) or other cardiac shunts. placed in a left lateral decubitus and Trendelenburg position.Sedation, hypothermia, and brain dehydration treatment were administered to the patient.
Day 2—24 h	patient remained hemodynamically stable	continued sedation with propofol and administered methylprednisolone and mannitol to reduce cerebral edema
Day 3	Magnetic Resonance Imaging (MRI) of the brain performed.	brain MRI showed swelling in the bilateral frontal and parietal cortex, indicating ischemic and hypoxic brain
Day 4	Patient's consciousness recovered	Patient were sensitive to light reflex, upper limb muscle strength returned to normal, and lower limb muscle strength returned to 2–3 levels
Day 11 (Discharge)	Discharged from hospital to a rehabilitation facility.	Patient was ambulatory with a cane and had mild residual left-sided weak

## Disscussion

Venous air embolism is a known complication associated with the insertion, maintenance, and removal of central venous catheters (CVCs). However, CAE following CVC removal remains a rare yet severe event, with reported mortality rates as high as 23% (5). Typically, air entering the venous circulation is filtered by the pulmonary capillary bed. However, in the presence of a right-to-left shunt, such as a patent foramen ovale (PFO), air can bypass this filtration, entering the systemic circulation and potentially causing embolisms in critical organs like the brain (6–8). This mechanism, known as paradoxical air embolism, is a well-documented pathway for CAE.

Notably, clinical observations indicate that some patients with CAE show no evidence of intracardiac shunting, suggesting alternative pathways. These may include a massive air burden overwhelming the pulmonary filter or the opening of bronchopulmonary anastomoses, allowing air to enter the pulmonary venous system directly. Notably, the case reported by Teifurova et al. highlights the potential for retrograde venous flow as another plausible mechanism for cerebral infarction in the absence of a shunt. Thus, this concept warrants greater clinical attention (7, 9, 10).

This case also highlights the potential therapeutic value of hypothermia in managing CAE. Standard management includes high-flow oxygen to promote nitrogen washout, hyperbaric oxygen therapy (HBO), and neuroprotective strategies (11, 12).

Although our patient did not receive HBO, significant neurological recovery was achieved through targeted temperature management (maintained at 34°C for 24 h, 0.25°C/h rewarming, bladder temperature monitoring) and measures to reduce intracranial pressure. This outcome suggests that therapeutic hypothermia may confer neuroprotection by reducing cerebral metabolic rate, mitigating oxidative stress, and suppressing inflammatory cascades (9, 11, 13–14). However, it is crucial to acknowledge the potential risks of therapeutic hypothermia, particularly during the rewarming phase. Rapid rewarming can lead to a significant increase in the number of cerebral gas microemboli. Therefore, we implemented a strictly controlled slow rewarming protocol (0.25°C per hour) to minimize the risk of emboli expansion and secondary brain injury potentially induced by overly rapid rewarming. Nevertheless, due to the rarity and acute nature of CAE, robust evidence to guide optimal treatment remains limited.

Finally, this case reinforces the critical importance of preventing CVC-related CAE, particularly in high-risk patients with postoperative delirium or impaired consciousness. Preventive strategies should include securing the catheter meticulously, employing appropriate sedation or physical restraints when necessary, and adhering strictly to standardized removal protocols—such as placing the patient in the Trendelenburg position and removing the catheter at end-exhalation (7, 15).

In conclusion, further research is essential to elucidate the non-shunt pathogenic mechanisms of CAE and to refine its treatment protocols, including the role of adjuvant therapies like hypothermia.

## Conclusion

A rare case of a patient with esophageal cancer who underwent successful recovery from CAE caused by CVC removal after surgery was reported. Through hypothermic brain protection and administered methylprednisolone and mannitol to reduce cerebral edema, the patient achieved a favorable prognosis. The study also analyzed the potential mechanisms of CAE, including the possibility of air embolism under intracardiac shunt and non-shunt conditions. Moreover, it highlights the critical role of hypothermia therapy in neuroprotection. Thus, this study provides important insights into the treatment and prevention of CAE, emphasizes the importance of standardized operation and timely intervention, and lays the foundation for future research.

## Data Availability

The raw data supporting the conclusions of this article will be made available by the authors, without undue reservation.
